# Analysis of the correlation and predictive performance of GNRI, CONUT, and PNI with MAFLD in Chinese adult physical examination population

**DOI:** 10.3389/fnut.2026.1726212

**Published:** 2026-04-21

**Authors:** Zhen Cheng, Siyue He, Chunyu Hu, Liang Wang, Laixi Zhang, Yuanling Tao, Li Sun, Zongtao Chen

**Affiliations:** Health Management Centre, First Affiliated Hospital of Army Medical University, Chongqing, China

**Keywords:** controlled nutritional status, geriatric nutritional risk index, metabolic-associated fatty liver disease, nutritional status, prognostic nutritional index

## Abstract

**Background:**

Metabolic-associated fatty liver disease (MAFLD) has become a major global health issue. Although nutrition is known to be associated with MAFLD, indices such as the Geriatric Nutritional Risk Index (GNRI), Controlling Nutritional Status (CONUT), and Prognostic Nutritional Index (PNI) have been used for prognostic assessment in diseases like cancer and heart disease, but their relevance and predictive performance in MAFLD, especially across different subgroups in China, still need validation.

**Methods:**

This study is a retrospective cross-sectional study conducted at the First Affiliated Hospital of the Army Medical University in China. A total of 10,901 cases were included (MAFLD group: 2,426 cases; non-MAFLD group: 8,475 cases). Logistic regression analysis was performed using SPSS 27.0 and R 4.5.1 to determine relevant associations (calculating OR values and 95% confidence intervals). Receiver operating characteristic (ROC) curves were plotted for the three nutritional indices—GNRI, CONUT, and PNI—to predict the occurrence of MAFLD in different sex, BMI, and age groups, and area under the curve (AUC), sensitivity, and specificity were calculated. Stratified analysis was performed to study the interaction between MAFLD and different subgroups.

**Results:**

There is a dose–response relationship between GNRI, CONUT, and PNI and MAFLD. In multivariable models, the risk of developing MAFLD in the fourth quartile of GNRI and PNI was 7.01 times (OR = 7.01, 95% CI: 5.08–9.67) and 1.82 times (OR = 1.82, 95% CI: 1.51–2.19) that of the first quartile, respectively Overall, the predictive performance of GNRI was superior to that of CONUT (AUC = 0.909) and PNI (AUC = 0.909), especially in women, individuals with BMI less than 24, and those under 35 years of age. Stratified analysis showed that in individuals under 35 years old without hyperlipidemia and without hyperglycemia, the association between these three nutritional indicators and MAFLD was stronger.

**Conclusion:**

GNRI, CONUT, and PNI can all serve as effective indicators for assessing the risk of MAFLD, among which GNRI has the highest application value, especially suitable for female, young people, and individuals with normal weight, and can provide new insights for the clinical screening and intervention of MAFLD.

## 1 Introduction

NAFLD has become an important global health problem, with a disease burden affecting approximately 25% of the population, and its prevalence is rapidly rising in both developed and developing countries, becoming a major cause of liver disease burden in many countries and regions (1). With the in-depth understanding of NAFLD, in early 2022, an international expert group consisting of 30 experts from 22 countries proposed to rename non-alcoholic fatty liver disease (NAFLD) to Metabolism-Associated Fatty Liver Disease (MAFLD) (2).

In recent years, the prevalence of MAFLD has been getting younger year by year, and it can progress to hepatitis, cirrhosis, liver cancer, etc., which is seriously harmful to health (3). How to detect and treat MAFLD in an early and timely manner is an important means to delay the progression of the disease (4). The pathogenesis of MAFLD is unclear and involves various factors such as genetics, environment, and lifestyle. Previous studies have shown a relationship between MAFLD and nutrition, and diets rich in saturated fats, trans fats, simple sugars, and animal proteins are harmful to liver health, promote lipid accumulation, and lead to metabolic disorders in MAFLD (5–8). However, the link between nutritional status and MAFLD risk has not been thoroughly investigated (9, 10).

The Geriatric Nutrition Risk Index (GNRI) was originally designed as an assessment tool to assess the nutritional status of older adults as a predictive marker of malnutrition (11). Lower GNRI values indicate a higher risk of malnutrition. A meta-analysis showed that lower GNRI is an independent predictor of all-cause mortality and major cardiovascular events in older patients with heart failure (12). However, in metabolic diseases, patients often exhibit higher levels of body mass index, and it is unclear whether higher GNRI levels are associated with adverse outcomes in these patients. Controlled nutritional status (CONUT) can comprehensively reflect the nutritional and inflammatory status of the body by comprehensively considering indicators such as serum albumin, total cholesterol, and lymphocyte count (13, 14). Previous studies have verified that CONUT can be used to evaluate the prognostic effect of tumor surgery. The Prognostic Nutrient Index (PNI) is commonly used to assess an individual’s immunonutritional status. PNI has been found to be useful in predicting mortality and cardiovascular risk in patients who are obese or have metabolic problems (15–17).

Nutritional indices such as CONUT, GNRI, and PNI cover factors including immune and nutritional status, as well as chronic inflammation, and have been shown to be prognostic markers for a range of diseases such as cancer, autoimmune diseases, and heart disease (5, 18, 19). Given that MAFLD is associated with low-grade inflammation, malnutrition, and immune dysfunction, the potential link between these factors and the prevalence of MAFLD is worth investigating (20). Examination of nutritional status can provide further understanding of the relationship between nutrition and NAFLD (21).

Although several studies have explored the relationship between various nutritional indices and the progression of NAFLD, given the regional differences in the epidemiology of MAFLD, this study aims to further verify the correlation between nutritional indices and MAFLD by screening the Chinese physical examination population, and evaluate and compare its predictive effect on the risk of MAFLD, aiming to provide new ideas for the prevention and diagnosis of MAFLD.

## 2 Methods

**Figure 1:**
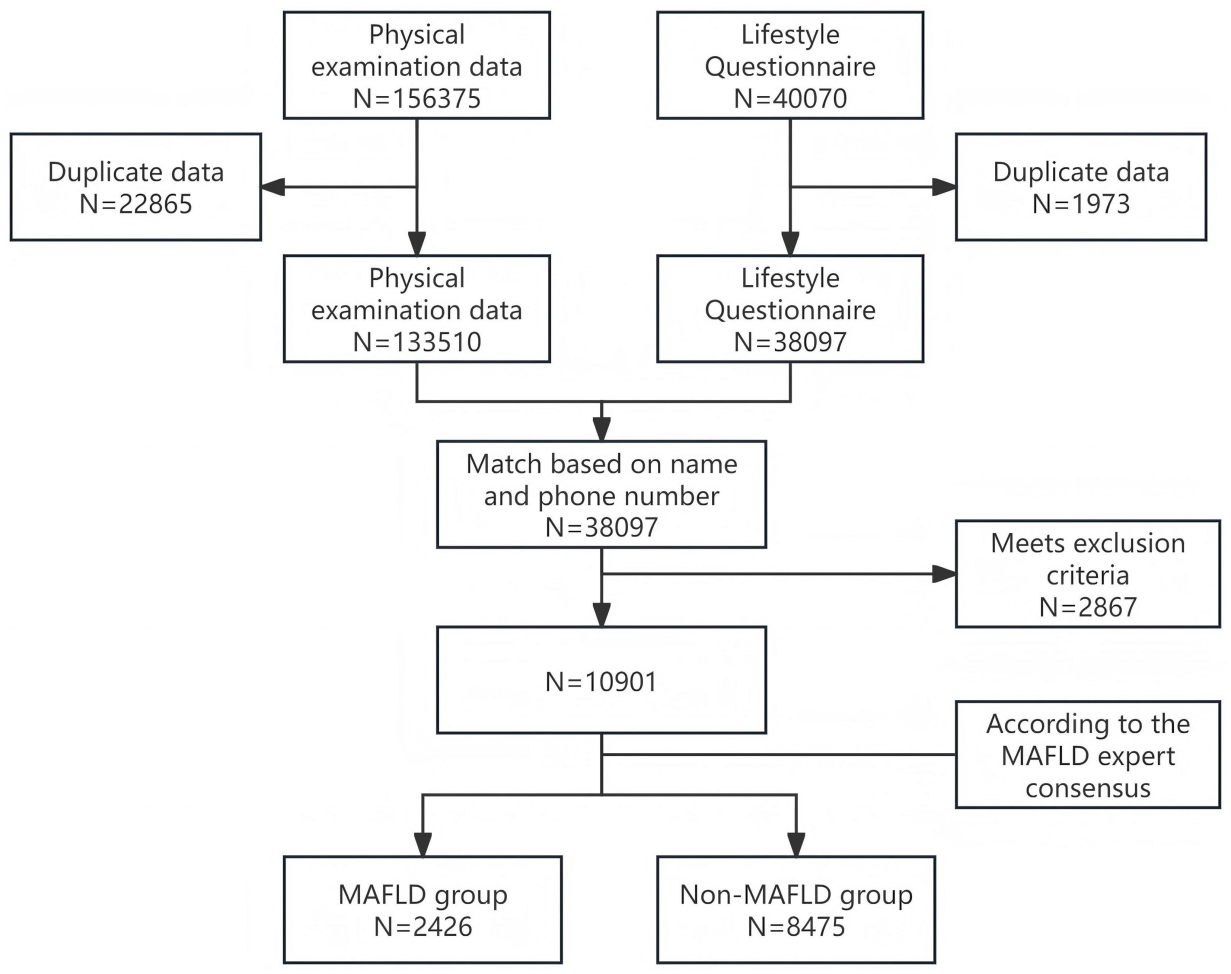
Research data screening flowchart.

This study was a retrospective cross-sectional study conducted at the First Affiliated Hospital of the Army Medical University in China from January 2021 to June 2023. Inclusion criteria were age ≥18 years; complete physical examination data, including basic information, biochemical indicators, and abdominal ultrasound results. Exclusion criteria were liver diseases with other clear causes, such as viral hepatitis (e.g., hepatitis B, hepatitis C), autoimmune liver disease, and drug-induced liver injury; malignant tumors; and severe dysfunction of major organs such as the heart, lungs, or kidneys. Initially, data from 156,375 subjects who underwent health examinations were collected. After removing duplicates and incomplete data and through rigorous screening, a total of 10,901 subjects were finally included, with 2,426 in the MAFLD group and 8,475 in the non-MAFLD group (Figure 1).

### 2.1 Diagnostic criteria for MAFLD

According to the diagnostic criteria in the “Guidelines for the Prevention and Treatment of Metabolic Associated (Non-Alcoholic) Fatty Liver Disease (2024 Edition)” issued by the Liver Disease Branch of the Chinese Medical Association: (1) hepatic steatosis indicated by abdominal ultrasound examination; (2) meeting at least one of the following metabolic risk factors: overweight or obesity (Body Mass Index BMI ≥ 24 kg/m^2^), excessive waist circumference (male waist ≥90 cm, female waist ≥85 cm), triglycerides (TG) ≥ 1.70 mmol/L, reduced high-density lipoprotein cholesterol (HDL-C) (male HDL-C < 1.04 mmol/L, female HDL-C < 1.30 mmol/L), abnormal blood glucose (fasting blood glucose ≥6.1 mmol/L or 2-h post-load glucose ≥7.8 mmol/L or previously diagnosed diabetes) (2).

### 2.2 Data collection and definition

Subjects underwent a physical examination the next morning while fasting. All physical examination items are participated and measured by professionally trained medical staff. The subjects did not wear shoes or thick clothes, and naturally stood on the physical examination equipment (device name and model: Intelligent Health Examination All-in-one Machine HW-900A, Henan Lejia Electronic Technology Co., Ltd., China) to measure height, weight, and body mass index (BMI). Systolic blood pressure (SBP) and diastolic blood pressure (DBP) are measured using an electronic sphygmomanometer (device name and model: HBP-9021, Omron Electronics LLC, Ltd., Japan) in the resting state. Blood specimens were obtained through the median cubital vein and the following indicators were detected by chemical analysis instrument (device name and model: BECKMAN COULTER Chemistry Analyzer AU5800, Beckman Kurt Co., Ltd., United States), alanine transferase (ALT), aspartate transferase (AST), glutamyl transferase (GGT), alkaline phosphatase (ALP), low-density lipoprotein cholesterol (LDL-C), High-density lipoprotein cholesterol (HDL-C), triglycerides (TG), total cholesterol (TC), fasting plasma glucose (FPG), albumin (Alb). The automatic hematology analyzer (equipment name and model: Auto hematology analyzer BC-6800, Shenzhen Mindray Bio-Medical Electronics Co., Ltd., China) was used to detect hemoglobin (HGB), white blood cell count (WBC), lymphocyte count (LYC), neutrophil count (NEU), monocyte count (MON), platelet count (PLT) and other indicators. Basic information such as gender, age, smoking history, and alcohol consumption history were collected through lifestyle questionnaires.

Hypertension: Systolic blood pressure >140 mmHg, diastolic blood pressure >90 mmHg. Hyperlipidemia: TC ≥ 6.2 mmol/L; LDL-C ≥ 4.1 mmol/L; TG ≥ 2.3 mmol/L; HDL-C < 1.0 mmol/L. hyperglycemia is defined according to the aforementioned abnormal blood glucose levels. Smoking history: Non-smoker; Former smoker (not smoking for more than 1 month); Smoker (smoking continuously for more than 1 year, more than 1 cigarette per day). Alcohol consumption history: Non-drinker; Former drinker (abstinent for more than 6 months); Drinker (drinking on average more than once per week).

### 2.3 Nutritional assessment index calculation

Geriatric Nutrition Risk Index (GNRI): calculated as GNRI = 1.489 × serum albumin (g/L) + 41.7 × (actual weight/ideal weight). Among them, the ideal weight (kg) for men = 0.75 × height (cm) −62.5, and the ideal weight (kg) for women = 0.60 × height (cm) -40. According to the GNRI value, the nutritional risk was divided into GNRI ≥98 as normal nutrition, 82 ≤ GNRI <97.9 as mild nutritional risk, 66 ≤ GNRI <81.9 as moderate nutritional risk, and GNRI <66 as severe nutritional risk (11).

Controlled nutritional status score (CONUT): Calculated by three laboratory parameters: serum albumin, lymphocyte count, and total cholesterol. The specific scoring criteria are: serum albumin level ≥3.50 g/dL is 0 points, 3.00–3.49 g/dL is 2 points, 2.50–2.99 g/dL is 4 points, and <2.50 g/dL is 6 points; lymphocyte count ≥1.6 × 10^9^/L scored 0 points, (1.2–1.599) × 10^9^/L scored 1 point, (8.0–1.199) × 10^9^/L scored 2 points, <8.00 × 10^9^/L scored 3 points; Total cholesterol ≥180 mg/dL is 0 points, 140–179 mg/dL is 1 point, 100–139 mg/dL is 2 points, and <100 mg/dL is 3 points. The three scores were summed to obtain the total CONUT score, with 0–1 being normal nutrition, 2–4 being mildly malnourished, 5–8 being moderately malnourished, and ≥9 being severely malnourished (22).

Prognostic nutrient index (PNI): Calculated as PNI = serum albumin (g/L) + 5 × lymphocyte count (×10^9^/L). According to the PNI value, the nutritional status was divided into PNI ≥ 50 as good nutrition, 40 ≤ PNI < 50 as mild malnutrition, 30 ≤ PNI < 40 as moderate malnutrition, and PNI < 30 as severe malnutrition (10).

### 2.4 Statistical methods

All data were statistically analyzed using SPSS27.0 software and R software version 4.5.1. Continuous variables were described as X ± S or M (P25 ~ P75) according to whether they were normally distributed, and the t-test or Mann-Whitney *U* rank-sum test was used for comparison between groups. Categorical variables were described as frequencies and percentages, and the 2 test was used for comparison between groups. They were divided into four groups according to the GNRI and PNI quartile levels, and two groups according to the median CONUT, and logistic regression was used to analyze the OR value and 95% CI of the risk of MAFLD under different correction conditions. The Receiver operator characteristiccurve (ROC) was plotted for predicting the occurrence of MAFLD under different genders, BMI levels, and age groups, and the areaunder curve (AUC), sensitivity, specificity, maximum Yorden index and corresponding cut-off values were calculated. Stratified analyses further examined the relationship between MAFLD and various demographic and health-related factors, including gender, age, marital status, ethnicity, smoking, alcohol consumption, BMI, hypertension, Hyperlipidemia, and hyperglycemia. The interaction between NAFLD and these factors was assessed using the *p*-value of the interaction term. Both were two-sided tests, and *p* < 0.05 was considered to be statistically significant.

## 3 Results

### 3.1 Comparison of general characteristics and biochemical indicators between two groups

A total of 10,901 data entries were collected, including 4,912 males (45.06%) and 5,989 females (57.94%), with a relatively even distribution between genders. The MAFLD group included 2,426 individuals, while the non-MAFLD group included 8,475 individuals. The MAFLD group (42.98 ± 11.0) had a higher average age compared to the non-MAFLD group (37.10 ± 10.91). Han ethnicity, married individuals, those who drink alcohol, smokers, and people with hyperlipidemia, hypertension, or hyperglycemia had a higher proportion of MAFLD. The MAFLD group had higher levels of age, GNRI, PNI, BMI, SBP, DBP, Alb, LYC, TC, NEU, PLT, MON, ALT, AST, GGT, ALP, TG, LDL-C, FPG, HGB, and WBC, and lower levels of CONUT and HDL-C, compared to the non-MAFLD group, with all differences being statistically significant (*p* < 0.05) (Table 1).

**Table 1:** Basic characteristics of participants according to the MAFLD and Non-MAFLD group.

Characteristics	Non-MAFLD group (*N* = 8,475)	MAFLD group (*N* = 2,426)	Total (*N* = 10,901)	*t*/*z*/χ^2^	*p*-value
Gender					
Female	5,378 (49.33%)	611 (5.60%)	5,989 (54.94%)	1115.939	<0.001
Male	3,097 (28.41%)	1,815 (16.65%)	4,912 (45.06%)		
Marital status					
No	2,008 (18.42%)	272 (2.50%)	2,280 (20.92%)	177.634	<0.001
Yes	6,467 (59.32%)	2,154 (19.76%)	8,621 (79.08%)		
Ethnic					
Han Chinese	8,267 (75.84%)	2,389 (21.92%)	10,656 (97.75%)	7.411	0.006
Ethnic minorities	208 (1.91%)	37 (0.34%)	245 (2.25%)		
Smoking					
Non Smoker	6,958 (63.83%)	1,486 (13.63%)	8,444 (77.46%)	470.932	<0.001
Former Smoker	328 (3.01%)	187 (1.72%)	515 (4.72%)		
Current Smoker	1,189 (10.91%)	753 (6.91%)	1,942 (17.81%)		
Drinking					
Non-Drinker	5,633 (51.67%)	1,062 (9.74%)	6,695 (61.42%)	479.29	<0.001
Former Drinker	878 (8.05%)	273 (2.50%)	1,151 (10.56%)		
Current Drinker	1,964 (18.02%)	1,091 (10.01%)	3,055 (28.02%)		
Age	37.10 ± 10.91	42.98 ± 11.10	38.41 ± 11.22	−23.078	<0.001
GNRI	108.97 ± 6.03	117.56 ± 6.61	110.89 ± 7.12	−57.512	<0.001
CONUT	0.98 ± 0.93	0.60 ± 0.81	0.90 ± 0.91	18.514	<0.001
PNI	53.57 ± 3.61	54.84 ± 3.97	53.85 ± 3.73	−14.11	<0.001
BMI	22.60 ± 2.85	27.17 ± 3.02	23.61 ± 3.46	−68.625	<0.001
SBP (mmHg)	117.28 ± 14.54	129.61 ± 16.42	120.02 ± 15.83	−33.431	<0.001
DBP (mmHg)	72.95 ± 10.40	82.03 ± 11.80	74.97 ± 11.37	−34.31	<0.001
Alb (g/L)	44.59 ± 2.40	44.91 ± 2.47	44.66 ± 2.42	−5.645	<0.001
LYC (10^9^/L)	1.80 ± 0.49	1.99 ± 0.57	1.84 ± 0.52	−14.914	<0.001
TC (mmol/L)	4.82 ± 0.90	5.28 ± 0.98	4.92 ± 0.94	−20.566	<0.001
NEU (10^9^/L)	3.33 ± 1.05	3.74 ± 1.08	3.42 ± 1.07	−16.729	<0.001
PLT (10^9^/L)	220.23 ± 52.13	224.37 ± 55.12	221.15 ± 52.84	−3.302	0.001
MON (10^9^/L)	0.31 ± 0.10	0.36 ± 0.11	0.32 ± 0.11	−16.219	<0.001
ALT (IU/L)	20.70 ± 17.40	39.63 ± 28.19	24.91 ± 21.77	−31.396	<0.001
AST (IU/L)	22.27 ± 12.49	28.65 ± 14.28	23.69 ± 13.18	−19.927	<0.001
GGT (IU/L)	23.95 ± 29.88	52.43 ± 56.78	30.29 ± 39.39	−23.773	<0.001
ALP (IU/L)	68.60 ± 21.07	80.43 ± 21.35	71.23 ± 21.70	−24.13	<0.001
TG (mmol/L)	1.17 ± 0.83	2.43 ± 1.79	1.45 ± 1.24	−33.583	<0.001
LDL-C (mmol/L)	2.94 ± 0.67	3.35 ± 0.68	3.03 ± 0.69	−26.625	<0.001
HDL-C (mmol/L)	1.42 ± 0.30	1.17 ± 0.24	1.36 ± 0.31	41.819	<0.001
FPG (mmol/L)	5.41 ± 0.83	6.16 ± 1.76	5.58 ± 1.15	−20.21	<0.001
HGB (g/L)	142.12 ± 15.50	154.24 ± 14.07	144.82 ± 16.01	−36.539	<0.001
WBC (10^9^/L)	5.60 ± 1.36	6.27 ± 1.47	5.75 ± 1.41	−20.261	<0.001
Hyperlipidemia					
No	7,184 (65.90%)	573 (5.26%)	7,757 (71.16%)	3436.234	<0.001
Yes	1,291 (11.84%)	1,853 (17.00%)	3,144 (28.84%)		
Hypertension					
No	7,721 (70.83%)	1,694 (15.54%)	9,415 (86.37%)	725.192	<0.001
Yes	754 (6.92%)	732 (6.71%)	1,486 (13.63%)		
Hyperglycemic					
No	7,857 (72.08%)	1,705 (15.64%)	9,562 (87.72%)	880.513	<0.001
Yes	618 (5.67%)	721 (6.61%)	1,339 (12.28%)		

Geriatric nutrition risk index (GNRI), Controlled nutritional status (CONUT), Prognostic nutrient index (PNI), body mass index (BMI), systolic blood pressure (SBP), diastolic blood pressure (DBP), alanine transferase (ALT), aspartate transferase (AST), glutamyl transferase (GGT), alkaline phosphatase (ALP), low-density lipoprotein cholesterol (LDL-C), High-density lipoprotein cholesterol (HDL-C), triglycerides (TG), total cholesterol (TC), fasting plasma glucose (FPG), albumin (Alb), hemoglobin (HGB), white blood cell count (WBC), lymphocyte count (LYC), neutrophil count (NEU), monocyte count (MON), platelet count (PLT).

### 3.2 Relationship between different body mass index levels and MAFLD

According to the quartiles of GNRI and PNI, they were each divided into four groups, and CONUT was divided into two groups based on the median. The Cramer’s V coefficients between GNRI, CONUT, PNI, and MAFLD were 0.488, 0.137, and 0.126, respectively, indicating that there is a certain dose–response relationship between the three nutritional indices and MAFLD, meaning that the prevalence of MAFLD increases as the levels of each index group increase (Table 2).

**Table 2:** Correlation between different nutritional index levels and MAFLD.

Index	Non-MAFLD group			MAFLD group			Cramer’s V	*p*-value
	*N*	MAFLD (%)	Total (%)	*N*	MAFLD (%)	Total (%)		
GNRI								
Q1	2,670	31.50%	24.50%	66	2.70%	0.60%	0.489	<0.001
Q2	2,487	29.30%	22.80%	236	9.70%	2.20%		
Q3	2,094	24.70%	19.20%	626	25.80%	5.70%		
Q4	1,224	14.40%	11.20%	1,498	61.70%	13.70%		
CONUT								
M1	6,233	73.50%	57.20%	2,122	87.50%	19.50%	0.137	<0.001
M2	2,242	26.50%	20.60%	304	12.50%	2.80%		
PNI								
Q1	2,281	26.90%	20.90%	457	18.80%	4.20%	0.126	<0.001
Q2	2,208	26.10%	20.30%	526	21.70%	4.80%		
Q3	2,103	24.80%	19.30%	611	25.20%	5.60%		
Q4	1,883	22.20%	17.30%	832	34.30%	7.60%		

Geriatric nutrition risk index (GNRI), Controlled nutritional status (CONUT), Prognostic nutrient index (PNI).

### 3.3 Univariate and multivariate logistic regression analysis

Univariate logistic regression analysis showed that male sex, alcohol consumption history, smoking history, higher age, BMI, GNRI, CONUT, PNI, and biochemical index levels such as SBP, DBP, Alb, LYC, TC, NEU, PLT, MON, ALT, AST, GGT, ALP, TG, LDL-C, FPG, HGB, WBC were all risk factors for the risk of MAFLD. Ethnic minorities and higher levels of CONUT and HDL-C are protective factors for MAFLD. Model 3 showed that the probability of MAFLD in the fourth group of GNRI and PNI was 7.01 times (OR: 7.01, 95% CI: 5.08, 9.67) and 1.82 times (OR: 1.82, 95% CI: 1.51, 2.19), respectively, and the probability of MAFLD in the fourth group was 0.61 times that of the first group (OR: 0.61, 95% CI: 0.52, 0.72) (Tables 3, 4).

**Table 3:** Univariate logistic regression analysis of the risk of developing MAFLD.

Characteristics	*B*	SE	Wald χ^2^	OR (95%CI)	*p*-value
Gender	1.64	0.05	998.22	5.16 (4.66, 5.71)	<0.001
Age	0.05	0.00	487.87	1.05 (1.04, 1.05)	<0.001
Marital status	0.90	0.07	168.87	2.46 (2.15, 2.82)	<0.001
Ethnic	−0.49	0.18	7.27	0.62 (0.43, 0.88)	0.007
Smoking			447.87		<0.001
Former Smoker	0.98	0.10	104.65	2.67 (2.21, 3.22)	<0.001
Current Smoker	1.09	0.06	395.74	2.97 (2.66, 3.30)	<0.001
Drinking			458.95		<0.001
Former Drinker	0.50	0.08	42.28	1.65 (1.42, 1.92)	<0.001
Current Drinker	1.08	0.05	458.84	2.95 (2.67, 3.25)	<0.001
GNRI	0.21	0.01	1901.79	1.24 (1.23, 1.25)	<0.001
CONUT	−0.53	0.03	320.32	0.59 (0.55, 0.62)	<0.001
PNI	0.09	0.01	211.76	1.09 (1.08, 1.11)	<0.001
BMI	0.51	0.01	2076.41	1.68 (1.64, 1.71)	<0.001
SBP	0.05	0.00	952.23	1.05 (1.05, 1.05)	<0.001
DBP	0.07	0.00	1016.38	1.08 (1.07, 1.08)	<0.001
Alb	0.06	0.01	32.73	1.06 (1.04, 1.08)	<0.001
LYC	0.68	0.04	245.02	1.97 (1.81, 2.15)	<0.001
TC	0.51	0.03	416.87	1.66 (1.58, 1.74)	<0.001
NEU	0.34	0.02	261.29	1.40 (1.34, 1.46)	<0.001
PLT	0.00	0.00	11.58	1.00 (1.00, 1.00)	0.001
MON	3.40	0.21	266.69	29.89 (19.88, 44.93)	<0.001
ALT	0.05	0.00	1065.56	1.05 (1.05, 1.05)	<0.001
AST	0.05	0.00	432.09	1.05 (1.05, 1.06)	<0.001
GGT	0.03	0.00	756.27	1.03 (1.02, 1.03)	<0.001
ALP	0.02	0.00	497.62	1.02 (1.02, 1.03)	<0.001
TG	1.09	0.03	1331.06	2.99 (2.82, 3.17)	<0.001
LDL-C	0.86	0.04	607.81	2.36 (2.20, 2.53)	<0.001
HDL-C	−3.46	0.11	1090.95	0.03 (0.03, 0.04)	<0.001
FPG	0.68	0.03	476.36	1.98 (1.86, 2.11)	<0.001
HGB	0.05	0.00	985.39	1.06 (1.05, 1.06)	<0.001
WBC	0.33	0.02	404.94	1.39 (1.34, 1.43)	<0.001

Geriatric nutrition risk index (GNRI), Controlled nutritional status (CONUT), Prognostic nutrient index (PNI), body mass index (BMI), systolic blood pressure (SBP), diastolic blood pressure (DBP), alanine transferase (ALT), aspartate transferase (AST), glutamyl transferase (GGT), alkaline phosphatase (ALP), low-density lipoprotein cholesterol (LDL-C), High-density lipoprotein cholesterol (HDL-C), triglycerides (TG), total cholesterol (TC), fasting plasma glucose (FPG), albumin (Alb), hemoglobin (HGB), white blood cell count (WBC), lymphocyte count (LYC), neutrophil count (NEU), monocyte count (MON), platelet count (PLT).

**Table 4:** Multivariate logistic analysis of the relationship between various nutritional indexes and MAFLD.

Characteristics	Model 1					Model 2					Model 3				
	B	SE	Wald χ^2^	OR (95% CI)	*p*-value	B	SE	Wald χ^2^	OR (95% CI)	*p*-value	B	SE	Wald χ^2^	OR (95% CI)	*p*-value
GNRI Q1			1420.77		<0.001			1416.64		<0.001			242.19		<0.001
GNRI Q2	1.20	0.15	67.76	3.32 (2.50, 4.42)	<0.001	1.21	0.15	68.91	3.36 (2.52, 4.47)	<0.001	0.67	0.16	18.49	1.96 (1.44, 2.65)	<0.001
GNRI Q3	2.21	0.14	260.86	9.13 (6.98, 11.94)	<0.001	2.23	0.14	264.53	9.30 (7.11, 12.16)	<0.001	0.99	0.16	40.15	2.69 (1.98, 3.66)	<0.001
GNRI Q4	3.69	0.14	727.48	39.84 (30.48, 52.08)	<0.001	3.70	0.14	732.28	40.63 (31.07, 53.13)	<0.001	1.95	0.16	140.15	7.01 (5.08, 9.67)	<0.001
CONUT M2	−0.82	0.07	136.22	0.44 (0.38, 0.50)	<0.001	−0.80	0.07	128.41	0.45 (0.39, 0.52)	<0.001	−0.50	0.09	33.72	0.61 (0.52, 0.72)	<0.001
PNI Q1			166.35		<0.001			165.08		<0.001			46.45		<0.001
PNI Q2	0.28	0.08	13.30	1.33 (1.14, 1.55)	<0.001	0.28	0.08	12.63	1.32 (1.13, 1.54)	<0.001	0.12	0.09	1.61	1.13 (0.94, 1.36)	0.205
PNI Q3	0.54	0.08	48.21	1.71 (1.47, 1.99)	<0.001	0.53	0.08	46.78	1.70 (1.46, 1.98)	<0.001	0.33	0.09	12.19	1.39 (1.16, 1.67)	<0.001
PNI Q4	0.96	0.08	148.99	2.60 (2.23, 3.03)	<0.001	0.95	0.08	147.60	2.59 (2.22, 3.02)	<0.001	0.60	0.10	38.93	1.82 (1.51, 2.19)	<0.001

Geriatric nutrition risk index (GNRI), Controlled nutritional status (CONUT), Prognostic nutrient index (PNI). Model 1: Adjust for gender (female coded as 0, male coded as 1), age, and ethnicity (Han coded as 0, minority coded as 1), using the first group of each nutritional index as a reference to measure the OR values of MAFLD risk at different levels of each index; Model 2: Based on Model 1, further adjust for alcohol consumption history, smoking history, and marital status; Model 3: Based on Model 2, further adjust for BMI, hyperlipidemia, hypertension, and hyperglycemia.

### 3.4 ROC curve analysis of the risk of MAFLD occurrence predicted by various nutritional indices

**Figure 2:**
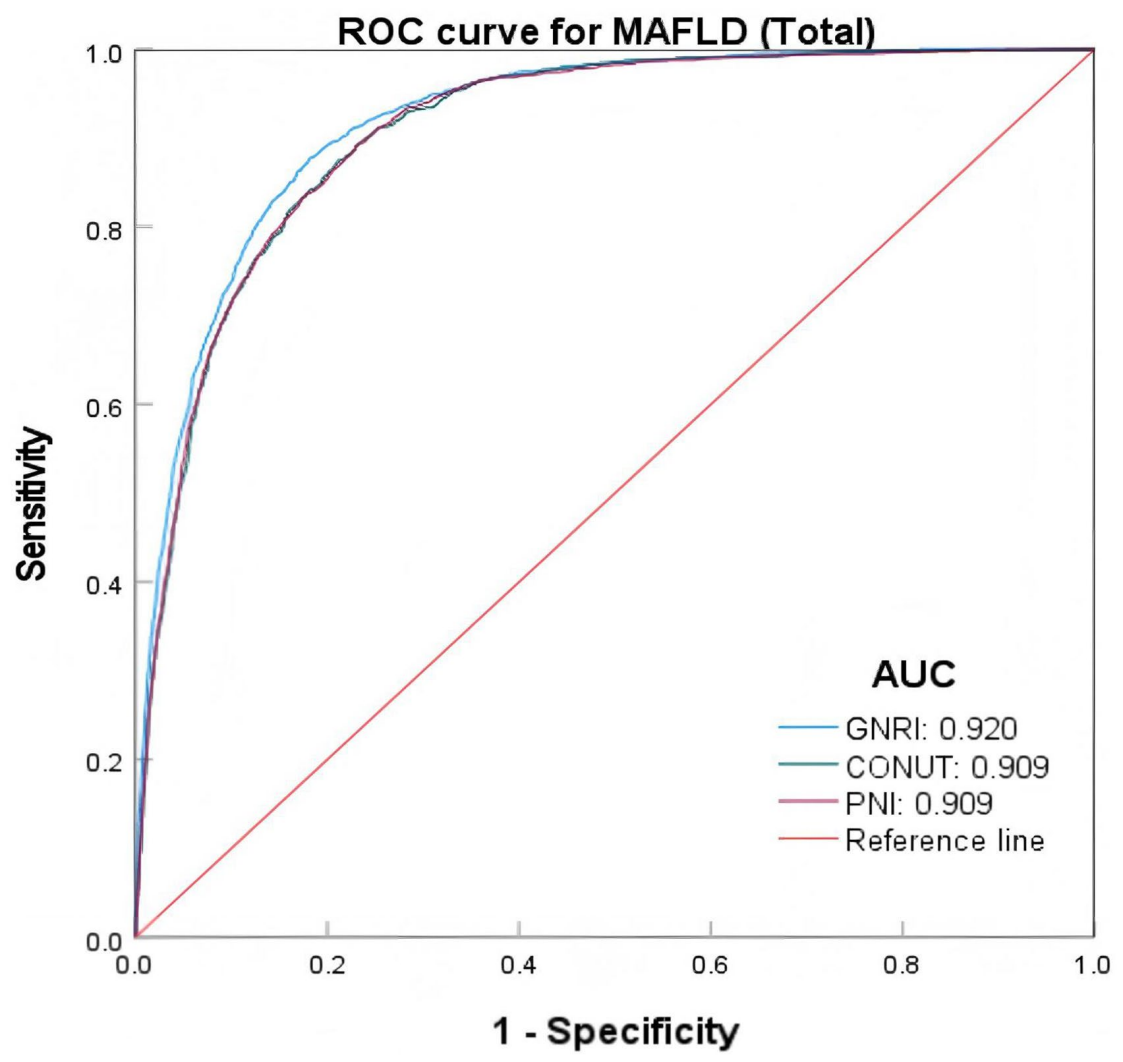
ROC curves of various nutritional indices predicting MAFLD risk.

In the total sample, the area under the receiver operating characteristic curve (AUC) of the model combining demographic, clinical variables, and GNRI was 0.920 (95% CI: 0.914, 0.925), with a sensitivity of 0.878 and a specificity of 0.818. The areas under the ROC curves (AUROC) for CONUT and PNI were 0.909 (95% CI: 0.903, 0.915), with sensitivities of 0.875 and 0.891, and specificities of 0.891 and 0.771, respectively (Table 5; Figure 2).

**Table 5:** Curve analysis of nutritional indexes predicting the risk of MAFLD.

Characteristics	Subgroup	AUC (95% CI)	*p*-value	Sensitivity	Specificity	Youden index	*z*	*p*-value
Total								
GNRI		0.920 (0.914, 0.925)	<0.001	0.878	0.818	0.696		
CONUT		0.909 (0.903, 0.915)	<0.001	0.875	0.788	0.663		
PNI		0.909 (0.903, 0.915)	<0.001	0.891	0.771	0.662		
Gender subgroup								
GNRI	Male	0.867 (0.857, 0.877)	<0.001	0.840	0.736	0.576	10.042	<0.001
	Female	0.937 (0.928, 0.947)	<0.001	0.895	0.833	0.728		
CONUT	Male	0.847 (0.837, 0.858)	<0.001	0.760	0.778	0.538	9.770	<0.001
	Female	0.924 (0.913, 0.935)	<0.001	0.939	0.765	0.704		
PNI	Male	0.848 (0.837, 0.859)	<0.001	0.779	0.758	0.537	10.132	<0.001
	Female	0.927 (0.916, 0.938)	<0.001	0.917	0.792	0.709		
BMI subgroup								
GNRI	BMI < 24	0.899 (0.882, 0.917)	<0.001	0.789	0.869	0.658	6.343	<0.001
	BMI ≥ 24	0.831 (0.820, 0.843)	<0.001	0.763	0.762	0.525		
CONUT	BMI < 24	0.890 (0.870, 0.909)	<0.001	0.741	0.909	0.650	7.455	<0.001
	BMI ≥ 24	0.800 (0.787, 0.813)	<0.001	0.756	0.739	0.495		
PNI	BMI < 24	0.884 (0.863, 0.904)	<0.001	0.748	0.900	0.648	6.545	<0.001
	BMI ≥ 24	0.803 (0.790, 0.816)	<0.001	0.760	0.737	0.497		
Age subgroup								
GNRI	<35	0.934 (0.926, 0.942)	<0.001	0.878	0.853	0.731	5.680	<0.001
	≥35	0.901 (0.893, 0.909)	<0.001	0.856	0.798	0.654		
CONUT	<35	0.922 (0.913, 0.930)	<0.001	0.936	0.767	0.703	5.016	<0.001
	≥35	0.890 (0.881, 0.898)	<0.001	0.816	0.817	0.633		
PNI	<35	0.920 (0.911, 0.930)	<0.001	0.932	0.780	0.712	4.597	<0.001
	≥35	0.891 (0.882, 0.900)	<0.001	0.834	0.796	0.630		

Geriatric nutrition risk index (GNRI), Controlled nutritional status (CONUT), Prognostic nutrient index (PNI).

**Figure 3:**
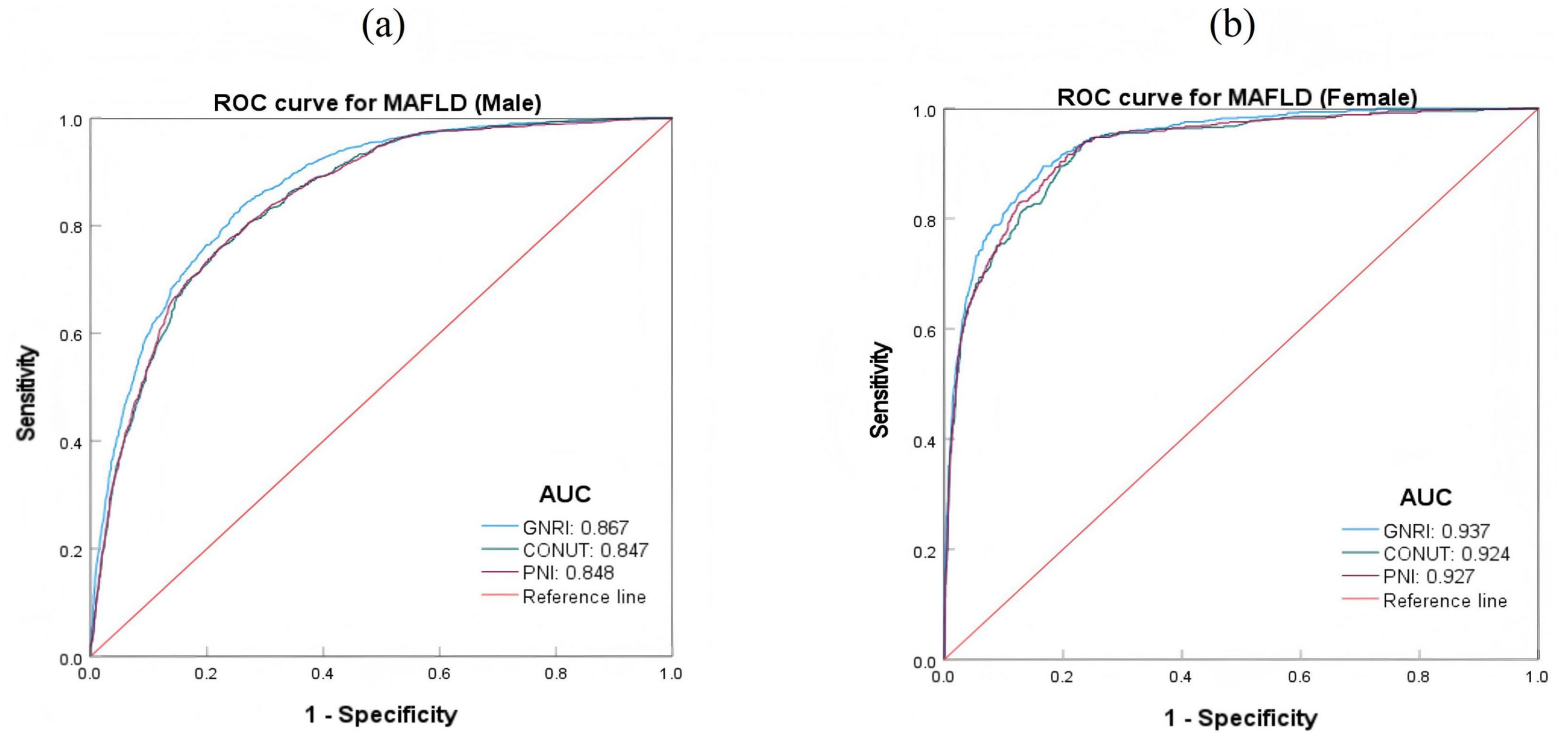
ROC curves for predicting MAFLD risk based on nutritional indices in different genders. **(A)** ROC curves for MAFLD (Male); **(B)** ROC curves for MAFLD (Female).

In different gender groups, after combining demographic and clinical variables, the plotted receiver operating characteristic curves showed that the predictive effect of the three nutritional indices GNRI, CONUT, and PNI was higher in females than in males. Among them, GNRI had a better predictive performance for MAFLD across gender groups compared to CONUT and PNI. The AUC value of GNRI in females was 0.937, while in males it was 0.867. The differences in predictive effects of CONUT and PNI for MAFLD across genders were not significant (Table 5; Figure 3).

**Figure 4:**
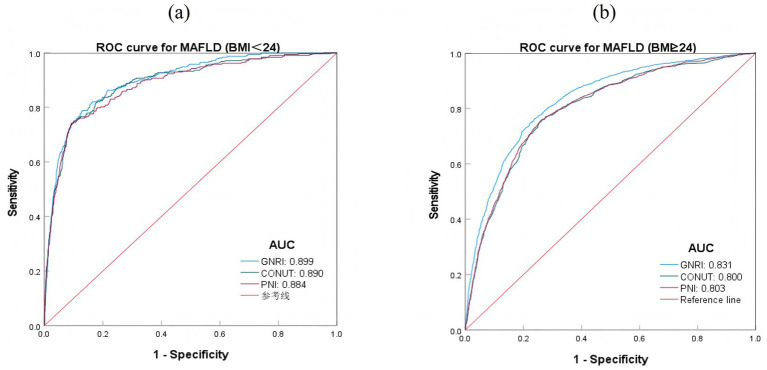
ROC curves of nutritional index predicting MAFLD risk at different BMI levels. **(A)** ROC curves for MAFLD (BMI < 24); **(B)** ROC curves for MAFLD (BMI > 24).

In different BMI groups, the predictive effects of the three nutritional indices GNRI, CONUT, and PNI were higher in the BMI < 24 group compared to the BMI ≥ 24 group (*p* < 0.05). In the BMI < 24 group, their AUC values were 0.899, 0.890, and 0.884, respectively, and there were no obvious differences in predictive performance among the three indices. In the BMI ≥ 24 group, GNRI had a better predictive effect for MAFLD compared to CONUT and PNI, while the difference between CONUT and PNI was not significant (Table 5; Figure 4).

**Figure 5:**
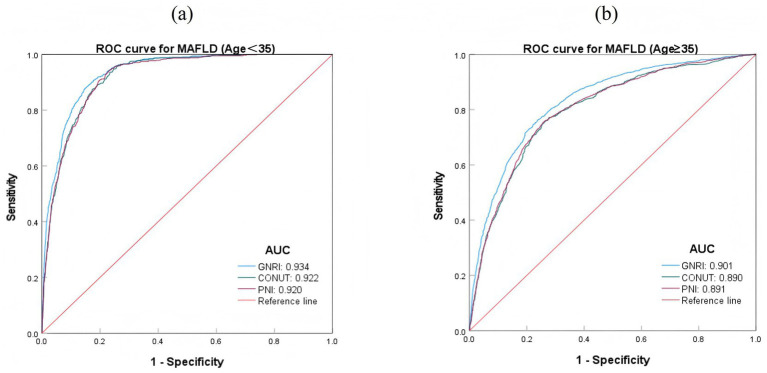
ROC curves of predicted MAFLD risk based on nutritional index for different age groups. **(A)** ROC curves for MAFLD (Age <35); **(B)** ROC curves for MAFLD (Age ≥35).

In different age groups, the predictive effects of the three nutritional indices GNRI, CONUT, and PNI were higher in the age <35 group compared to the age ≥35 group (*p* < 0.05). Among them, GNRI had a better predictive effect for MAFLD than CONUT and PNI across different age groups. The AUC value of GNRI was 0.934 in the age <35 group and 0.901 in the age ≥35 group. The predictive effects of CONUT and PNI across different age groups did not show significant differences (Table 5; Figure 5).

### 3.5 Stratified analysis

**Figure 6:**
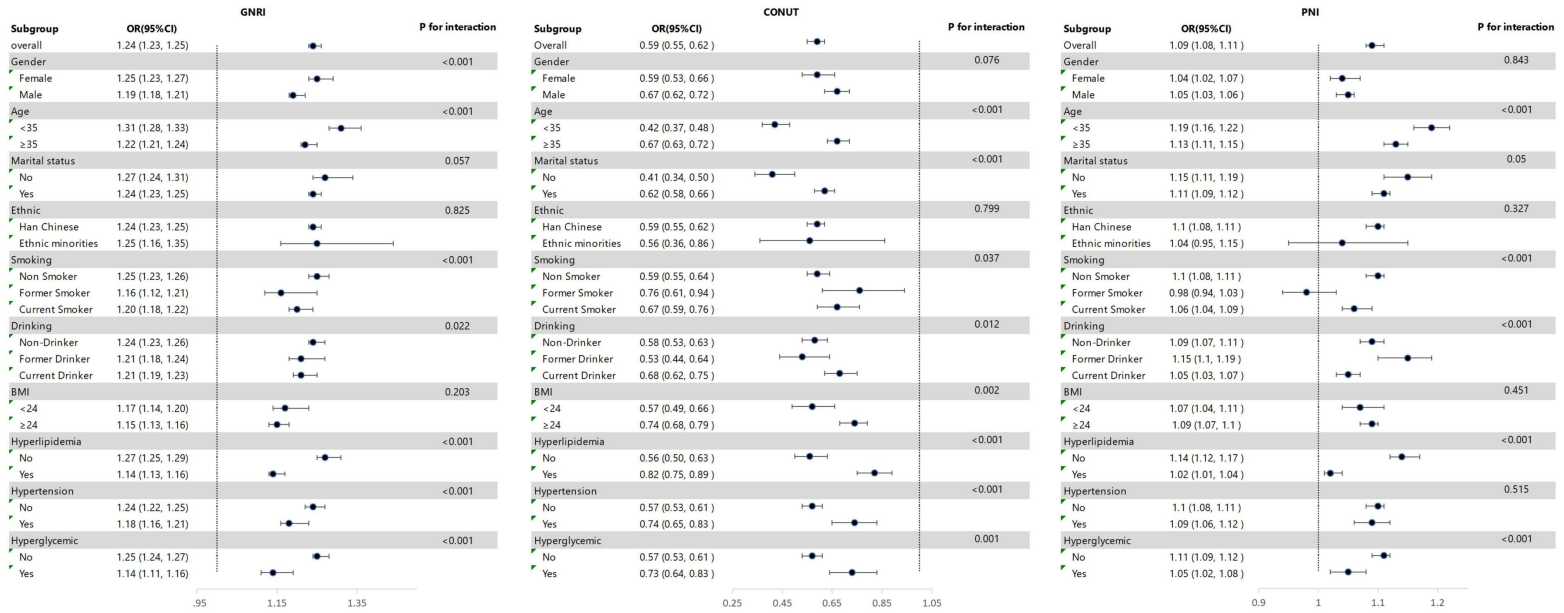
Association between nutritional index and MAFLD in each subgroup.

The GNRI, CONUT, and PNI all showed significant interactions with age, smoking, drinking, hyperlipidemia, and hyperglycemia in the incidence of MAFLD. The associations between these three nutritional indices and MAFLD were stronger in individuals under 35 years old, without hyperlipidemia, and without hyperglycemia. Additionally, the GNRI score also had significant interactions with gender (*P*_interaction_ <0.001) and hypertension (*P*_interaction_ <0.001) in the incidence of MAFLD, with stronger associations observed in women and individuals without hypertension. Furthermore, the CONUT score showed significant interactions with marital status (*P*_interaction_ <0.001), BMI (*P*_interaction_ = 0.002), and hypertension (*P*_interaction_ <0.001) in the incidence of MAFLD, with stronger associations observed in unmarried individuals, those with BMI < 24, and those without hypertension (Figure 6).

## 4 Discussion

In this study, the average age of the MAFLD group was higher than that of the non-MAFLD group. Additionally, the prevalence of MAFLD was higher among ethnic minorities, married individuals, those with a history of alcohol consumption and smoking, as well as people with hyperlipidemia, hypertension, and hyperglycemia. Previous studies have found that aging significantly increases the risk of liver fat accumulation, which may be related to age-associated declines in liver enzyme activity and reduced insulin sensitivity (23). Alcohol consumption and smoking, as classic risk factors for MAFLD, mainly aggravate hepatocellular steatosis by inducing oxidative stress in the liver and promoting the release of inflammatory factors such as TNF-*α* and IL-6 (24–26). Most MAFLD patients also have common metabolic comorbidities, such as obesity, hypertension, atherogenic dyslipidemia, or type 2 diabetes (27), which is highly consistent with conclusions from existing studies (27, 28).

In terms of biochemical indicators, the levels of BMI, SBP, DBP, TC, TG, LDL-C, ALT, AST, GGT and other indicators in the MAFLD group were significantly higher than those in the non-MAFLD group, and the HDL-C level was reduced, which is consistent with the pathophysiological characteristics of MAFLD (29). It is worth noting that the levels of GNRI, CONUT, and PNI in the MAFLD group were higher than those in the non-MAFLD group, suggesting that nutritional status was closely related to MAFLD. At the same time, combined with the quartile (GNRI, PNI) and median (CONUT) groupings, it was found that all three nutritional indices had a certain dose–response relationship with MAFLD, indicating that the proportion of MAFLD increased with the increase of the nutritional index grouping level. This result was further validated by univariate and multivariate logistic regression analysis, which showed that elevated GNRI, CONUT, and PNI were all risk factors for MAFLD. In the multivariate model 3 after adjusting for gender, age, ethnicity, lifestyle habits and metabolic indicators, the risk factors of GNRI and PNI were still significant, and the probability of MAFLD in the fourth group was 7.01 times and 1.82 times higher than that of the first group, respectively, while CONUT was shown as a protective factor. This difference is related to the different evaluation dimensions and methods of each nutritional index.

GNRI is mainly based on serum albumin and body weight changes, and its elevation usually reflects overnutrition, while long-term overnutrition can lead to a large accumulation of fat in the liver, and excessive lipid accumulation will exacerbate inflammation, oxidative stress, and organ structural damage caused by lipotoxicity (30), which in turn induces MAFLD. PNI is an effective indicator of immunonutritional status, and its increase is associated with an increase in the body’s nutrient reserves by serum albumin and lymphocyte counts. CONUT is also used as an index to comprehensively evaluate nutritional status and immune function, and its increase may reflect a certain state of inflammatory stress in the body. Unlike GNRI, lymphocyte count, as the second component of CONUT and PNI, can reflect immune function and certain levels of inflammation (31). In patients with MAFLD, chronic inflammation is an important driver of disease progression. Because overnutrition is associated with low-grade chronic inflammation, chronic inflammation increases the risk of metabolic and cardiovascular disease, promotes autoreactivity, and undermines protective immunity (32–34), thereby affecting the disease progression of fatty liver. The higher the CONUT score, the less likely there was to be overnutrition, which was consistent with the conclusion of the protective effect of CONUT in this study.

ROC curve analysis showed that in the overall sample, after combining demographic and clinical variables, the AUC value of GNRI predicting MAFLD was higher than that of CONUT and PNI, and GNRI had higher sensitivity and specificity. This indicates that GNRI has a superior performance in predicting MAFLD. In different gender groups, the predictive performance of the three nutritional indices was higher in females than in males, and the AUC value of GNRI in females (0.967) was significantly higher than in males (0.867). This may be related to gender-related differences in hormone levels, as estrogen in females can enhance the association between nutritional indices and liver fat accumulation by regulating liver lipid metabolism enzyme activity, thereby improving the predictive performance of nutritional indices (35, 36). In different BMI groups, the predictive performance of the three nutritional indices was higher in the BMI < 24 group than in the BMI ≥ 24 group, and in the BMI ≥ 24 group, the predictive performance of GNRI remained superior to CONUT and PNI. This suggests that nutritional status has a more significant impact on MAFLD in individuals with normal weight, whereas in overweight/obese populations, BMI as a major risk factor may partially mask the predictive role of nutritional indices. Studies have reported that metabolically unhealthy lean individuals may experience more ectopic fat accumulation, mainly manifested as visceral fat distribution. Compared with metabolically healthy non-obese patients, non-obese patients with metabolically unhealthy MAFLD face a higher risk of liver injury and cardiovascular disease (2, 37).

Among different age groups, the predictive performance of the three nutritional indices was significantly higher in the under-35 group compared to the 35-and-over group. This age-dependent difference may be attributed to age-related physiological and pathological changes. Younger individuals typically have better liver metabolic function, and the occurrence of MAFLD is more directly related to nutritional imbalance, allowing nutritional indices to accurately reflect liver lipid metabolism abnormalities. However, aging impairs liver metabolism and increases the prevalence of chronic comorbidities (such as type 2 diabetes) and the use of multiple medications, which may disrupt the intrinsic association between nutritional parameters and MAFLD, thereby weakening predictive power (38). The superior performance of GNRI may be due to its components—including serum albumin and the ratio of body weight to ideal body weight—which can directly reflect protein-energy status, an important regulator of liver synthetic function and lipid homeostasis (39). In contrast, CONUT and PNI include lymphocyte counts, and lymphocytes in older adults are susceptible to immunosenescence and chronic inflammation, which may reduce discriminative ability and obscure age-related differences (40). These results suggest that GNRI is the preferred nutritional index for MAFLD screening, particularly in younger populations.

Stratified analysis showed that GNRI, CONUT, and PNI were more strongly associated with MAFLD in individuals under 35 years of age, without hyperlipidemia, and without hyperglycemia. This indicates that in young people without metabolic abnormalities, nutritional status is a key factor in the occurrence of MAFLD, while unhealthy lifestyle habits such as smoking and drinking, as well as metabolic abnormalities, can alter the association between nutritional indices and MAFLD through cumulative effects (41–44). Furthermore, GNRI was more strongly associated in women and individuals without hypertension; CONUT was more strongly associated in unmarried individuals, those with a BMI < 24, and those without hypertension. Other studies have shown that lack of physical activity, prolonged sitting, and high intake of sugar, fat, and ultra-processed foods can increase the risk of fatty liver, while moderate-intensity exercise and intermittent fasting can reduce the risk of developing fatty liver (45–47). These results suggest that physiological characteristics, lifestyle conditions, and health statuses such as hypertension and BMI can influence the effect of nutritional indices on MAFLD. In clinical practice, it is necessary to combine population characteristics to use nutritional indices for targeted MAFLD risk assessment (39).

### 4.1 Limitations

This study has the following limitations: First, it is a cross-sectional study, which cannot determine the causal relationship between nutritional indices and MAFLD. Prospective cohort studies are needed in the future to further verify this. Second, the study did not include detailed lifestyle information such as dietary patterns and exercise habits, which may influence the association between nutritional indices and MAFLD.

## 5 Conclusion

GNRI, CONUT, and PNI can serve as effective indicators for assessing the risk of MAFLD, with GNRI having the highest application value. Its assessment is more effective in women, young people, and individuals with normal body weight. In clinical practice, population characteristics can be considered when using nutritional indices to screen for MAFLD risk, while adjusting nutritional status can provide new approaches for the prevention and intervention of MAFLD.

## Data Availability

The raw data supporting the conclusions of this article will be made available by the authors, without undue reservation.
